# Involving people and patients in health care, health policy and research – across the board

**DOI:** 10.1111/hex.12922

**Published:** 2019-05-31

**Authors:** Carolyn A. Chew‐Graham

**Affiliations:** ^1^ Professor of General Practice Research Research Institute, Primary Care and Health Sciences Faculty of Medicine and Health Sciences Keele University Keele Staffordshire UK

Welcome to all our readers for this bumper edition of *Health Expectations*. As regular readers will know, our journal ‘promotes critical thinking and informed debate about all aspects of public participation in health care and health policy’. This issue covers a breadth of topics around involving people and patients in health care, in health policy and in research.

A number of papers in this issue focus on *the illness*: Eassey and colleagues report a qualitative study, with interviews conducted with people with severe asthma, recruited from primary and specialist care, and explored the role of patient autonomy in the experience of living with, and managing, their condition. The authors suggest that autonomy extends beyond information seeking and shared decision making. Another manuscript from Australia reports the co‐production of a Dementia Support Kit, which aimed to provide understandable information for people with dementia, and their carers. Ferguson and colleagues report a qualitative study (again from Australia) in which interviews were conducted with men who had used the ambulance service for mental health, drug or alcohol problems. They highlight both positive and negative experiences reported by study participants and suggest the need for further training to enhance paramedics’ communication.

However, the *illness* cannot be seen in isolation, and the important context of the *consultation* and the *service* must be considered. Shared decision making^1^ is held up as a pre‐requisite for patient‐centred care.^2^ Brown and Salmon's thoughtful review article highlights the tension between ‘respect for self‐determination and a comparable respect for what it means to be a patient’ and suggests that practitioners have the responsibility to decide when it is, in fact, appropriate to *lead* decision making within the consultation. The importance of the communication in the consultation is explored by Houwen and colleagues in their cohort study, reporting patient expectations in consultations for medically explained and unexplained symptoms. Their study has a key message for primary care clinicians: the importance of exploring, and paying attention, to the patient's expectations. Patient priorities in people with a terminal illness (or their caregivers) were explored in the study reported by Lewis and colleagues. This study highlighted two barriers to delivering satisfactory, safe and patient‐centred end‐of‐life (EOL) care: the need for more skilful communication to be demonstrated by healthcare professionals, and the system within which EOL care is given, leaving patients and caregivers with unmet needs.

Other manuscripts focus on *services*. Lopatina describes a multi‐step approach to patient engagement in service re‐design; Mulvale and colleagues explore citizens’ involvement in co‐designing public services, identify challenges and suggest improvements, which should emphasize recognising and being responsive to the needs of specific groups. Mulvale included in their umbrella term of ‘vulnerable’ groups, adults with mental health problems, personality disorders, survivors of domestic violence and young offenders. Brangan's manuscript reports a qualitative study with people in more deprived areas, which aimed to learn lessons for increasing uptake of the *NHS health check*.

A number of manuscripts in this issue report the *participation in research* of seldom‐heard groups. Brangan's manuscript focused on minority ethnic groups and deprived areas; McDermott provides insight into the emotional, social and practical importance of research to people with multiple sclerosis and myalgic encephalomyelitis / chronic fatigue syndrome, suggesting a range of research topics for the future. How to engage young people in research is explored by Dovey‐Pearce, with lessons learned including the importance of building relationships, managing practical concerns as well as identifying and managing emotional needs which arise in younger people participating in research. The authors conclude, however, that young people can contribute usefully and positively to the research process.

HEX welcomes manuscripts which not only report patients and the public as participants, but where Patient and Public Involvement and Engagement has been key to the study (indeed, we require that authors state how PPIE has involved their research). INVOLVE^3^ gives valuable advice about how service users and members of the public can give valuable advice about defining the research question, conducting the research, analysing and interpreting data and dissemination of findings.

In summary, we have papers which report studies using a range of research methodologies, across a range of clinical conditions, health care systems and countries. Do enjoy this issue – I am sure you will find the range of articles stimulating, and I do hope that there is something in this edition for you.
